# Supplementary material for: Concurrent category-selective neural activity across the ventral occipito-temporal cortex supports a non-hierarchical view of human visual recognition
Source: eLife. 2026 Aug 4;15:RP109640. doi: 10.7554/eLife.109640 (PMC13436960; doi:10.7554/eLife.109640)
Supplement: Supplementary file 2 [file elife-109640-supp1.docx]

**Supplementary tables for :**

**Concurrent category-selective neural activity across the ventral occipito-temporal cortex supports a non-hierarchical view of human visual recognition**

Corentin Jacques^1^, Jacques Jonas^1,2^ , Sophie Colnat-Coulbois^3^, Bruno Rossion*^1,2^

1 Université de Lorraine, CNRS, IMoPA, F-54000 Nancy, France
2 Université de Lorraine, CHRU-Nancy, Service de Neurologie, F-54000 Nancy, France
3 Université de Lorraine, CHRU-Nancy, Service de Neurochirurgie, F-54000 Nancy, France

@mail: bruno.rossion@univ-lorraine.fr

**This file includes:**

Tables S1 - S4

|  | **Left hemisphere** | **Right hemisphere** |  |  |  |
| --- | --- | --- | --- | --- | --- |
| **VMO** | **6 (4)** | **3 (3)** |  |  |  |
| **IOG** | **19 (10)** | **46 (12)** |  |  |  |
| **medFG** | **7 (6)** | **7 (6)** |  |  |  |
| **latFG** | **58 (21)** | **65 (25)** |  |  |  |
| **MTG/ITG** | **7 (6)** | **2 (2)** |  |  |  |
| **antCOS** | **18 (13)** | **26 (16)** |  |  |  |
| **antFG** | **10 (9)** | **10 (8)** |  |  |  |
| **antOTS** | **63 (29)** | **57 (34)** |  |  |  |
| **antMTG/ITG** | **7 (6)** | **8 (6)** |  |  |  |
| **AMG** | **8 (6)** | **6 (6)** |  |  |  |

**Table A: Number of contacts showing significant face-selective activity increase in each anatomical region.** Number of contacts showing significant face-selective activity increase in each anatomical region. The corresponding number of participants in which these contacts were found is indicated in parenthesis. Acronyms: VMO: ventro-medial occipital cortex; IOG: inferior occipital gyrus; medFG: medial fusiform gyrus and collateral sulcus; latFG: lateral fusiform gyrus and occipito-temporal sulcus; MTG/ITG: inferior and middle temporal gyri; antCoS: anterior collateral sulcus; antOTS: anterior occipito-temporal sulcus; antFG: anterior fusiform gyrus; antMTG/ITG: anterior middle and inferior temporal gyri.

| **Latency method** | **Hemi** | **Region comparison** | **Median diff. (ms)** | **Cohen's d** | **pval. FDR** | **Nb Partcpts region 1** | **Nb Partcpts region 2** | **Equivalence Prop in ROPE (%)** | **Bayes factor Cauchy prior (>3)** |
| --- | --- | --- | --- | --- | --- | --- | --- | --- | --- |
| z-score | Left | OCC vs PTL | 17.6 | 0.32 | 0.9228 | 12 | 25 | 29 | 0.7 |
| delta slope | Left | OCC vs PTL | 7.8 | 0.19 | 0.9228 | 12 | 25 | 46 | 1.3 |
| Broken stick | Left | OCC vs PTL | 3.9 | 0.11 | 1 | 12 | 25 | 60 | 2.2 |
| % of peak | Left | OCC vs PTL | 5.9 | 0.15 | 0.98475789 | 12 | 25 | 54 | 1.8 |
| z-score | Left | PTL vs ATL | 2 | 0.04 | 1 | 25 | 40 | 58 | 2.4 |
| delta slope | Left | PTL vs ATL | 0 | 0 | 0.9228 | 25 | 40 | 82 | 11.6 |
| Broken stick | Left | PTL vs ATL | 9.1 | 0.15 | 0.93066667 | 25 | 40 | 57 | 2.4 |
| % of peak | Left | PTL vs ATL | 5.9 | 0.12 | 1 | 25 | 40 | 68 | 3.6 |
| z-score | Left | OCC vs ATL | 21.5 | 0.47 | 1 | 12 | 40 | 23 | 0.4 |
| delta slope | Left | OCC vs ATL | 5.9 | 0.03 | 0.9228 | 12 | 40 | 71 | 5.9 |
| Broken stick | Left | OCC vs ATL | 13.1 | 0.21 | 0.93066667 | 12 | 40 | 47 | 1.4 |
| % of peak | Left | OCC vs ATL | 9.8 | 0.2 | 0.9228 | 12 | 40 | 44 | 1.1 |
| z-score | Right | OCC vs PTL | 11.7 | 0.14 | 1 | 13 | 27 | 56 | 2.6 |
| delta slope | Right | OCC vs PTL | 3.9 | 0.02 | 0.9228 | 13 | 27 | 70 | 6 |
| Broken stick | Right | OCC vs PTL | -15.9 | 0.31 | 0.9228 | 13 | 27 | 61 | 2.7 |
| % of peak | Right | OCC vs PTL | -15.6 | 0.3 | 0.9228 | 13 | 27 | 71 | 4.2 |
| z-score | Right | PTL vs ATL | -39.1 | 0.56 | 0.9228 | 27 | 43 | 36 | 1.1 |
| delta slope | Right | PTL vs ATL | -17.6 | 0.11 | 0.9228 | 27 | 43 | 75 | 7.3 |
| Broken stick | Right | PTL vs ATL | -14.2 | 0.24 | 0.9228 | 27 | 43 | 71 | 4.4 |
| % of peak | Right | PTL vs ATL | -13.7 | 0.26 | 0.9228 | 27 | 43 | 72 | 4.4 |
| z-score | Right | OCC vs ATL | -27.3 | 0.42 | 0.9228 | 13 | 43 | 60 | 2.3 |
| delta slope | Right | OCC vs ATL | -15.6 | 0.6 | 0.9228 | 13 | 43 | 41 | 0.7 |
| Broken stick | Right | OCC vs ATL | -30.8 | 0.55 | 0.9228 | 13 | 43 | 58 | 2 |
| % of peak | Right | OCC vs ATL | -31.3 | 0.68 | 0.9228 | 13 | 43 | 48 | 1.3 |

**Table B** **: Statistical comparisons of face-selective onset latencies split by hemispheres** Statistical comparisons of face-selective onset latencies across regions (OCC vs PTL, PTL vs ATL, OCC vs ATL) in each hemisphere using permutation tests et equivalence testing (proportion of difference in ROPE and Bayes factor) for 4 different onset estimation methods. Table contains the median latency difference between regions (Median diff.), effect size (Cohen’s d) of the difference, p-value of the permutation test (pval. FDR), number of participants included in each region.

| **Latency method** | **Region comparison** | **Median diff. (ms)** | **Cohen's d** | **pval. FDR** | **Nb Partcpts region 1** | **Nb Partcpts region 2** | **Equivalence Prop in ROPE (%)** | **Bayes factor Cauchy prior (>3)** |
| --- | --- | --- | --- | --- | --- | --- | --- | --- |
| z-score | IOG vs latFG | 7.8 | 0.16 | 1 | 22 | 43 | 50 | 1.7 |
| delta slope | IOG vs latFG | 9.8 | 0.07 | 1 | 22 | 43 | 82 | 10.9 |
| Broken stick | IOG vs latFG | -1.4 | 0.03 | 1 | 22 | 43 | 79 | 6.1 |
| % of peak | IOG vs latFG | 0 | 0 | 1 | 22 | 43 | 85 | 10.1 |
| z-score | latFG vs antFG+ | -2 | 0.04 | 1 | 43 | 72 | 63 | 2.7 |
| delta slope | latFG vs antFG+ | -9.8 | 0.09 | 1 | 43 | 72 | 90 | 19.8 |
| Broken stick | latFG vs antFG+ | 4.4 | 0.05 | 1 | 43 | 72 | 79 | 7.5 |
| % of peak | latFG vs antFG+ | 0 | 0 | 1 | 43 | 72 | 87 | 12.8 |
| z-score | IOG vs antFG+ | 5.9 | 0.13 | 1 | 22 | 72 | 53 | 1.5 |
| delta slope | IOG vs antFG+ | 0 | 0 | 1 | 22 | 72 | 73 | 3.6 |
| Broken stick | IOG vs antFG+ | 2.8 | 0.03 | 1 | 22 | 72 | 84 | 9 |
| % of peak | IOG vs antFG+ | 0 | 0 | 1 | 22 | 72 | 89 | 12.8 |

**Table C** : **Statistical comparisons of face-selective onset latencies measured in HFB signal across core face-selective regions (IOG, latFG, antFG).** Statistical comparisons of face-selective onset latencies measured in HFB signal across core face-selective regions (IOG, latFG, antFG) using permutation tests et equivalence testing (proportion of difference in ROPE and Bayes factor) for 4 different onset estimation methods (same as in main). Table contains the median latency difference between regions (Median diff.), effect size (Cohen’s d) of the difference, p-value of the permutation test (pval. FDR), number of participants included in each region.

| **Latency method** | **Region comparison** | **Median diff. (ms)** | **Cohen's d** | **pval. FDR** | **Nb Partcpts region 1** | **Nb Partcpts region 2** | **Equivalence Prop in ROPE (%)** | **Bayes factor Cauchy prior (>3)** |
| --- | --- | --- | --- | --- | --- | --- | --- | --- |
| Broken stick | OCC_vs_PTL | 11.1 | 0.22 | 0.343125 | 41 | 88 | 47 | 1.5 |
| % of peak | OCC_vs_PTL | 0 | 0 | 0.876 | 41 | 88 | 89 | 13.2 |
| delta slope | OCC_vs_PTL | 15.6 | 0.23 | 0.1905 | 41 | 88 | 43 | 1.4 |
| Broken stick | PTL_vs_ATL | -15.7 | 0.31 | 0.004498 | 88 | 113 | 84 | 8.9 |
| % of peak | PTL_vs_ATL | -7.8 | 0.1 | 0.06075 | 88 | 113 | 100 | 666.9 |
| delta slope | PTL_vs_ATL | -29.3 | 0.33 | 0.004498 | 88 | 113 | 63 | 3.5 |
| Broken stick | OCC_vs_ATL | -5.2 | 0.09 | 0.1905 | 41 | 113 | 88 | 10.4 |
| % of peak | OCC_vs_ATL | -7.8 | 0.09 | 0.06075 | 41 | 113 | 100 | 3492.4 |
| delta slope | OCC_vs_ATL | -11.7 | 0.14 | 0.255857 | 41 | 113 | 83 | 8.9 |

**Table D : Statistical comparisons of face-selective onset latencies measured in low frequency event-related potentials.** Statistical comparisons of face-selective onset latencies measured in low frequency event-related potentials across regions (OCC vs PTL, PTL vs ATL, OCC vs ATL) using permutation tests et equivalence testing (proportion of difference in ROPE and Bayes factor) for 3 different onset estimation methods (same as in main text except for ‘z-score’ method, which is unusable with low frequencies). Table contains the median latency difference between regions (Median diff.), effect size (Cohen’s d) of the difference, p-value of the permutation test (pval. FDR), number of participants included in each region. Despite significantly later ATL than PTL onset latencies (with small effect sizes), the difference is still within bounds of equivalence when taking into account estimated axonal conduction delays between the two regions.
