# Supplementary material for: Concurrent category-selective neural activity across the ventral occipito-temporal cortex supports a non-hierarchical view of human visual recognition
Source: eLife. 2026 Aug 4;15:RP109640. doi: 10.7554/eLife.109640 (PMC13436960; doi:10.7554/eLife.109640)
Supplement: Peer review history

# Editor report: eLife Assessment

**DOI:** 10.7554/eLife.109640.3.sa0

**Authors:** Iris IA Groen

**Affiliations:** University of Amsterdam Netherlands

**Keywords:** Compelling, Important

This **important** study addresses a classic debate in visual processing, using a strong method applied to an impressive dataset obtained from a rare clinical population to evaluate hierarchical models of visual object perception. The paper provides **compelling** evidence that the hierarchical model is only partly supported: as expected, neural responses in ventral visual cortex show increased representational selectivity for faces along the posterior-anterior axes, but the onsets of the signals do not show a temporal hierarchy, indicating more parallel processing.

# Referee report: Reviewer #1 (Public review)

**DOI:** 10.7554/eLife.109640.3.sa1

Summary:

This manuscript aims to test the idea that visual recognition (of faces) is hierarchically organized in the human ventral occipital-temporal cortex (VOTC). The paper proposes that if VOTC has a hierarchical organization, this should be seen in two independent features of the VOTC signal. First, hierarchy assumes that signals along the hierarchy increase in representational complexity. Second, hierarchy assumes a progressive increase in the onset time of the earliest neural response at each level of the hierarchy. To test these predictions, the authors extract high-frequency broadband signals from iEEG electrodes in a very large sample of patients (N=140). They find that face selectivity in these signals is distributed across the VOTC with increasing posterior-anterior face selectivity, hence providing evidence for the first prediction. However, they also find broadband activity to occur concurrently, therefore challenging the view of a serial hierarchy.

Strengths:

(1) The hypothesis (that VOTC is hierarchically organized) and predictions (that hierarchy predicts increases in representational complexity and increases in onset time) were clearly described.

(2) The number of subjects sampled (140) is extremely large for iEEG studies that typically involve <10 subjects. Also, 444 face selective recording contacts provide a very nice sampling of the areas of interest.

Comments on revised version:

The authors have performed additional analyses and checks and I would now rate the findings as important and compelling.

# Referee report: Reviewer #2 (Public review)

**DOI:** 10.7554/eLife.109640.3.sa2

Summary:

This very ambitious project addresses one of the core questions in visual processing related to the underlying anatomical and functional architecture. Using a large sample of rare and high-quality EEG recordings in humans, the authors assess whether face-selectivity is organised along a posterior-anterior gradient, with selectivity and timing increasing from posterior to anterior regions. The evidence suggests that it is the case for selectivity, but the data are more mixed about the temporal organisation, which the authors use to conclude that the classic temporal hierarchy described in textbooks might be questioned, at least when it comes to face processing.

Strengths:

A huge amount of work went into collecting this highly valuable dataset of rare intracranial EEG recordings in humans. The work is worth publishing for the data alone, assuming they are shared in an easily accessible and documented format. Currently, the OSF repository linked in the article is empty, so no assessment of the data can be made. The topic is important and a key question in the field is addressed. The EEG methodology is strong, relying on a well-established and high SNR SSVEP method. The method is particularly well suited to clinical populations, leading to interpretable data in a few minutes of recordings. The authors have attempted to quantify the data in many different ways, and provided various estimates of selectivity and timing, with matching measures of uncertainty. Non-parametric confidence intervals and comparisons are provided, using resampling that preserve dependencies in a hierarchical manner, which is rare. Two types of analyses are also provided to support evidence in favour of the lack of practical significance for some of the comparisons. Collectively, the various analyses and rich illustrations provide convincing evidence in favour of the conclusions.

Comments on revised version:

The authors have addressed all my previous comments and the work is mostly limited by the lack of pre-registration and the exploratory nature of some of the analyses. However, with data and code available, other teams can assess the impact of researchers' degrees of freedom on the main outcomes.

# Author comment: Author response

**DOI:** 10.7554/eLife.109640.3.sa3

**Authors:** Corentin Jacques, Jacques Jonas, Sophie Colnat-Coulbois, Bruno Rossion

**Affiliations:** Ingénierie Moléculaire et Physiopathologie Articulaire Nancy France; Ingénierie Moléculaire et Physiopathologie Articulaire Nancy France; Université de Lorraine Nancy France; Ingénierie Moléculaire et Physiopathologie Articulaire Nancy France

The following is the authors’ response to the original reviews.

**Public Reviews:**

**Reviewer #1 (Public review):**

Summary:

This manuscript aims to test the idea that visual recognition (of faces) is hierarchically organized in the human ventral occipital-temporal cortex (VOTC). The paper proposes that if VOTC has a hierarchical organization, this should be seen in two independent features of the VOTC signal. First, hierarchy assumes that signals along the hierarchy increase in representational complexity. Second, hierarchy assumes a progressive increase in the onset time of the earliest neural response at each level of the hierarchy. To test these predictions, the authors extract high-frequency broadband signals from iEEG electrodes in a very large sample of patients (N=140). They find that face selectivity in these signals is distributed across the VOTC with increasing posterior-anterior face selectivity, hence providing evidence for the first prediction. However, they also find broadband activity to occur concurrently, therefore challenging the view of a serial hierarchy.

Strengths:

(1) The hypothesis (that VOTC is hierarchically organized) and predictions (that hierarchy predicts increases in representational complexity and increases in onset time) were clearly described.

(2) The number of subjects sampled (140) is extremely large for iEEG studies that typically involve <10 subjects. Also, 444 face selective recording contacts provide a very nice sampling of the areas of interest.

We would like to thank this reviewer for their positive comments and evaluation of our manuscript.

Weaknesses:

(1) A control analysis where areas have known differences in response onset should be performed to increase confidence that the proposed analyses would reveal expected results when a difference in response onset was present across areas. From Figure 3, it can be seen that many electrodes are placed in earlier visual areas (V1-V3) that have previously been shown to have earlier broadband responses to visual images compared to VOTC (e.g. Martin et al., 2019, JNeurosci https://doi.org/10.1523/JNEUROSCI.1889-18.2018). The same analyses as in Figures 4 and 5 should be used comparing VOTC to early visual areas to confirm that the analyses would detect that V1-V3 have earlier onsets compared to VOTC.

First, we would like to mention that the analyses performed in our paper are commonly accepted analyses to extract time-domain information.

Yet, the reviewer is right that, considering our claim, evidence that our approach does indeed allow revealing ‘true’ differences in onset latencies would provide further support for our claims. The solution proposed by the reviewer is interesting but the number of face-selective recording contacts/sites in posterior occipital cortex (colored disks in figure 3) is too small for any meaningful comparison. Moreover, while absolute responses to visual images should indeed emerge earlier in early visual cortex than association cortex, it may not be the case for category-selective responses to visual images (which is what our claim is about).

To address the reviewer’s concern, we used face-selective responses from regions known to have different response onset latencies: occipital and posterior temporal lobe vs. medial temporal lobe structures (e.g., Mormann et al., 2008, https://pmc.ncbi.nlm.nih.gov/articles/PMC2676868/). Waveforms and onset latencies for these regions (OCC, PTL, MTL) are shown in Author response image 1. Despite the small number of contacts showing significant face-selective activity in the MTL (N=20) and the lower SNR in this region, the onset latency differences between OCC/PTL and MTL are significant using all 4 methods of latency estimation (see methods in the main manuscript), with medium to large effect sizes. This was despite noisy latency estimates for the MTL (in particular, the ‘delta slope’ method could not be used to get meaningful Cohen’s d when comparing MTL to OCC). Latency estimates are also slightly higher for the ‘% of peak’ method than in the manuscript, as we estimated the latency at 25% of the peak (instead of 20% in the manuscript), again to allow meaningful estimations for the MTL.

**Author response image 1.** 

In addition to this, we performed a simulation analysis where we statistically compared the measured PTL signals to ATL signals that have been artificially, incrementally, shifted forward in time. Author response image 2 shows (top row) the measured onset latencies differences between the 2 regions (PTL minus ATL) estimated using 4 different approaches as a function of the temporal shift applied to ATL, as well as the associated p-values (bottom row). As shown in Author response image 2, the ‘original’ unshifted data yields no significant difference between the 2 regions. The difference however becomes significant when ATL signals is shifted forward in time 10 to 30 ms, depending on the method used.

**Author response image 2.** 

These 2 observations provide evidence that our approach does indeed allow revealing ‘true’ differences in onset latencies, further supporting our claims.

Last, as also suggested by reviewer 2, we conducted a thorough equivalence testing using ROPE and Bayesian factor to support the lack of differences between regions. Equivalence bounds and region of practical equivalence (ROPE) were defined to account for physiological variability corresponding to a small effect (Cohen’s d = 0.199, i.e. standard in equivalence testing) and axonal conduction delays between regions (i.e. ATL is further away from early visual cortex than OCC). These analyses, now reported in the result section of the revised manuscript (Table 1), largely confirm the hypothesis of concurrent onset latencies across VOTC.

(2) It is unclear why correlating mean timeseries helps understand how much variance is shared between regions (Figure 4). Any variance between images is lost when averaging time series across all images, and this metric thus overestimates the variance shared between areas. Moreover, the finding that correlating time domain signals across VOTC areas does not differ from correlating signals within an area could be driven by this averaging. For example, if the same analysis was done on electrodes in left and right V1 when half of the images had contrast in the left hemifield and the other half had contrast in the right hemifield, the average signals may correlate extremely well, while this correlation falls apart on a trial-by-trial basis. These analyses therefore need to be evaluated on a trial-by-trial basis.

This is an interesting comment. We agree that variance between images is lost when averaging time-series across all images. However, to use the reviewer’s analogy, in order to support the claim that left and right V1 would show the same onset times and time-course (i.e., no hemispheric lateralization) for lateralized presentations (=the same kind of claim that we make in our paper), it’s the average response across images that should be compared, not a correlation run on a trial-by-trial basis (which would indeed falls apart because of a lack of response in the ipsilateral V1).

Moreover, we would like to emphasize that the goal of this analysis in our paper is not to make claims about the variance shared between regions. In fact, this is not a key analysis in our paper, the outcome of which is not strictly necessary for the main argument made. Finally, if we were to perform a (time-consuming) image-by-image analysis in our study, correlations would be weak due to low signal-to-noise ratio (each face image appears only 1.6 times per stimulation sequence on average) and the fact that each face image appears after a different non-face image across presentations.

(3) Previous studies on visual processing in VOTC have shown that evoked potentials are more predictive of the onset of visual stimuli than broadband activity (e.g. Miller et al., 2016, PLOS CB, https://doi.org/10.1371/journal.pcbi.1004660). Testing the prediction from a hierarchical representation that signals along the VOTC increase in onset time should therefore include an evaluation of evoked potential onsets in addition to broadband signals.

We have used HFB responses in our study as these signals tend to be easier to characterize in the time domain than evoked potentials, and they are more local given their reduced SNR compared to evoked potentials (Jacques et al., 2022; https://pmc.ncbi.nlm.nih.gov/articles/PMC9457683/). Moreover we have previously shown highly correlated time courses across HFB and low frequency evoked potentials in the same paradigm (Jacques et al., 2022, eLife).

Yet, to address this reviewer’s concern, we replicated the main analyses on low-frequency event-related potential signal, identifying contacts exhibiting significant face-selective responses in the same manner as in Jacques et al (2022). Namely, we start from bipolar-referenced sequences of recording corresponding to the full visual stimulation sequences (~70 s). For each recorded intracerebral contact, we average sequences in the time-domain, crop the average to contain an integer number of face frequency (1.2 Hz) cycles, run an FFT on the cropped sequences and identify the significant contacts with a Z-score procedure identical to that used for HFB signals. We then notch-filter out the visual response (6 Hz and harmonics) from the full length sequences, extract short epochs from the filtered sequences around the onset of each face image, average across epoch for each recording contact, subtract the mean signal measured in the baseline (-0.166 to 0 s relative to face onset) and take the absolute value (to be able to average across contacts despite differences of morphology and polarity). Significant contacts are then subjected to the same analyses as for the HFB signal.

Results from these analyses are presented as supplementary material (Figure S9, Table S4) in the revised manuscript (referenced in lines of the main manuscript). While we were not able to obtain reliable latency estimates using the z-score method with the same parameters as for HFB signal, these analyses with ERP signal indicate similar onset latencies for ERPs as for HFB activity and largely replicate observations made with HFB. In particular, onset latencies were in a very similar range (~100 to 140 ms) with similar patterns across regions or along VOTC and between-region signal correlations. There were also a few significant face-selective responses over posterior ventro-medial occipital cortex, likely overlapping ‘early visual cortex’ (V1,V2v,V3v,hV4), probably due to limited low-level contributions in this paradigm (see Or et al., 2019, JOV; here). Over these regions, onset latency was systematically earlier (up to 40ms) than in slightly more anterior regions, (i.e. anterior to -80 mm) where very little variability in onset latency was found up to the ATL region. We have acknowledged this in the revised manuscript.

(4) Testing the second prediction, that the onset time of processing increases along the VOTC posterior to anterior path, is difficult using the iEEG broadband signal, because from a signal processing perspective, broadband signals are inherently temporally inaccurate, given that they are filtered. Any filtering in the signal introduces a certain level of temporal smoothing. The manuscript should clearly describe the level of temporal smoothing for the filter settings used.

The reviewer is right that HFB signals are temporally smoothed, potentially yielding slightly underestimated onset latencies. However, our time-frequency analyses parameters ensured a minimal degree of smoothing. In fact, the original submission already contained a description of the expected temporal smoothing resulting from the wavelet transform. This is what we wrote in the original submission: “The number of cycles (i.e., central frequency) of the wavelet was adapted as a function of frequency from 2 cycles at the lowest frequency to 9 cycles at the highest frequency. The temporal smoothing resulting from the wavelet transform was minimal: wavelets had 20 ms of full width at half maximum (FWHM) across the frequency range (i.e. median of FWHM computed at each frequency bin), ensuring that onset timing information is accurate up to 10 ms (i.e half of the FWHM).”

In the revised manuscript we further elaborate as follows:

“The number of cycles (i.e., central frequency) of the wavelet was adapted as a function of frequency from 2 cycles at the lowest frequency to 9 cycles at the highest frequency. The temporal smoothing resulting from the wavelet transform was minimal: wavelets had a temporal spread of 20 ms (full width at half maximum - FWHM) across the frequency range (i.e. median of FWHM computed at each frequency bin). A simulation of HFB signals with a constant abrupt onset time and realistic signal-to-noise ratio indicates that the potential underestimation of onset latency due to the wavelet analysis is around 5-10 ms, which is on par with the value of the half width at half maximum (=FWHM/2 = 20/2 ms).”

Author response image 3 displays simulated HFB signal (using identical wavelet parameters than in our manuscript) in an ideal scenario with a response starting at 150 ms in all trials (N=150 trials), reaching maximum 10 ms later. This provides a theoretical estimate of the slight underestimation of onset latency due to the wavelet transform. It shows onset latency estimates are at most 12 ms underestimation of true onset time.

**Author response image 3.** 

That being said, given the physiological noise in the data, the fact that the response onset likely varies slightly from trial to trial, with a variable slope in activity increase, these wavelet parameters (within a certain margin) have likely little influence on the actual latency estimation.

(5) The onsets of neural activity in VOTC are surprisingly early: around 80-100 ms. This is earlier than what has previously been reported. For example, the cited Quian Quiroga et al. (2023) found single neuron responses to have the earlier onset around 125 ms (their Figure 3). Similarly, the cited Jacques et al., 2016b and Kadipasaoglu et al., 2017 papers also observe broadband onsets in VOTC after 100 ms. Understanding the temporal smoothing in the broadband signal, as well as showing that typical evoked potentials have latencies compared to other work, would increase confidence that latencies are not underestimated due to factors in the analysis pipeline.

In the revised manuscript, as suggested by reviewer 2, we have modified the data resampling strategy (using hierarchical bootstrap and permutation test that respects the nested structure of the data) to estimate onset latencies, confidence interval and permutation tests. Moreover, since the absolute onset latency estimates depend on the methods used, we now provide estimates using 4 different methods. The overall absolute onset latencies differ slightly across the 4 methods but all median onset latencies vary between 95 ms and 130 ms, which is similar to what was reported in some of the participants in Kadipasaoglu et al., 2017 (https://journals.plos.org/plosone/article?id=10.1371/journal.pone.0188834; note that latencies reported at the level of single sites or individual are usually higher due to lower signal-to-noise ratio). Moreover, these latencies for face-selective responses are actually very similar to those measured for non-selective/absolute responses to visual stimuli (e.g. Jacques et al., 2016: around 90-100 ms for faces in FG; Yoshor et al., 2007: ~100 ms in posterior Fusiform Gyrus; Regev et al. 2018: 90-120ms in posterior to middle FG). Other studies, measuring non-selective responses using more ‘conservative’ onset detection methods, find slightly later onset latencies (e.g. Cao et al. 2025: 139 ms in posterior FG; Martin et al., 2019: ~150 ms in ventral occipital -VO- regions).

Cao R, Zhang J, Zheng J, Wang Y, Brunner P, Willie JT, Wang S. 2025. A neural computational framework for face processing in the human temporal lobe. Current Biology. DOI: https://doi.org/10.1016/j.cub.2025.02.063

Martin AB, Yang X, Saalmann YB, Wang L, Shestyuk A, Lin JJ, Parvizi J, Knight RT, Kastner S. 2019. Temporal dynamics and response modulation across the human visual system in a spatial attention task: An ECoG study. Journal of Neuroscience 39:333–352. DOI: https://doi.org/10.1523/JNEUROSCI.1889-18.2018, PMID: 30459219

Regev TI, Winawer J, Gerber EM, Knight RT, Deouell LY. 2018. Human posterior parietal cortex responds to visual stimuli as early as peristriate occipital cortex. European Journal of Neuroscience 48:3567–3582. DOI: https://doi.org/10.1111/ejn.14164, PMID: 30240547

Yoshor D, Bosking WH, Ghose GM, Maunsell JHR. 2007. Receptive fields in human visual cortex mapped with surface electrodes. Cerebral Cortex 17:2293–2302. DOI: https://doi.org/10.1093/cercor/bhl138, PMID: 17172632

As an important note, in the revised manuscript, we have removed data from 3 recording contacts from 1 participant that were located in the upper bank of the Calcarine Sulcus, which is actually outside of our VOTC region of interest. The 3 contacts being located in dorsal V1 or V2 were showing very early responses and were biasing our latency estimates for the OCC region.

(6) Understanding the extent to which neural processing in the VOTC is hierarchical is essential for building models of vision that capture processing in the human brain, and the data provides novel insight into these processes.

For additional context, a schematic figure of the hierarchical view and a more parallel system described in the paragraph on models of visual recognition (lines 553) would help the reader interpret and understand the implications of the paper.

Our observations in the current study clearly indicates concurrent face-selective processing in the VOTC, which is incompatible with a serial hierarchical model. While we discuss how such concurrent activity could be implemented in the cortex (e.g., via direct input from ‘early visual cortex’ to different VOTC face-selective clusters), our data do not allow to provide more evidence in that respect to what already exists in the literature. Moreover, we are not providing data regarding connectivity (feedforward or re-entrant) either between face-selective regions or between these regions and ‘early visual cortex’.

Author response image 4 shows a very simplified versions of standard hierarchical/serial versus concurrent/parallel models.

**Author response image 4.**

**Reviewer #2 (Public review):**

Summary:

This very ambitious project addresses one of the core questions in visual processing related to the underlying anatomical and functional architecture. Using a large sample of rare and high-quality EEG recordings in humans, the authors assess whether face-selectivity is organised along a posterior-anterior gradient, with selectivity and timing increasing from posterior to anterior regions. The evidence suggests that it is the case for selectivity, but the data are more mixed about the temporal organisation, which the authors use to conclude that the classic temporal hierarchy described in textbooks might be questioned, at least when it comes to face processing.

Strengths:

A huge amount of work went into collecting this highly valuable dataset of rare intracranial EEG recordings in humans. The data alone are valuable, assuming they are shared in an easily accessible and documented format. Currently, the OSF repository linked in the article is empty, so no assessment of the data can be made. The topic is important, and a key question in the field is addressed. The EEG methodology is strong, relying on a well-established and high SNR SSVEP method. The method is particularly well-suited to clinical populations, leading to interpretable data in a few minutes of recordings. The authors have attempted to quantify the data in many different ways and provided various estimates of selectivity and timing, with matching measures of uncertainty. Non-parametric confidence intervals and comparisons are provided. Collectively, the various analyses and rich illustrations provide superficially convincing evidence in favour of the conclusions.

We thank the reviewer for their positive comments on our manuscript.

Weaknesses:

(1) The work was not pre-registered, and there is no sample size justification, whether for participants or trials/sequences. So a statistical reviewer should assess the sensitivity of the analyses to different approaches.

Pre-registration of fundamental research in a clinical context is quite uncommon for intracranial data, owing, for instance to the time needed to accumulate data, or to the uncertainty of cortical sampling location in a given participant. Nevertheless, in the current study, sample size is much higher than in typical intracranial studies (usually 5-20 participants), in fact much higher than most typical Cognitive Neuroscience research. The same is true for the number of recording contacts (>10000 site here), and the number of trials considered for analysis. Each participant had a minimum of 164 face trials and an average of 262 trials (i.e. an average of 3.2 stimulation sequences of 82 trials), which is higher than most standard human electrophysiological studies.

In the revised manuscript, to unsure that we have the maximum available power, and because our hypothesis is independent of hemisphere, we collapsed data across hemispheres for all analyses. We nevertheless provide analyses split by hemispheres as supplementary material.

In addition, since onset latency estimations depend on the methods used, we now report onset latencies from 4 different methods (2 statistical and 2 non-statistical).

(2) Frequentist NHST is used to claim lack of effects, which is inappropriate, see for instance:

Greenland, S., Senn, S. J., Rothman, K. J., Carlin, J. B., Poole, C., Goodman, S. N., & Altman, D. G. (2016). Statistical tests, P values, confidence intervals, and power: A guide to misinterpretations. European Journal of Epidemiology, 31(4), 337-350. https://doi.org/10.1007/s10654-016-0149-3

Rouder, J. N., Morey, R. D., Verhagen, J., Province, J. M., & Wagenmakers, E.-J. (2016). Is There a Free Lunch in Inference? Topics in Cognitive Science, 8(3), 520-547. https://doi.org/10.1111/tops.12214

Please see reply to the next comment (3).

(3) In the frequentist realm, demonstrating similar effects between groups requires equivalence testing, with bounds (minimum effect sizes of interest) that should be pre-registered:

Campbell, H., & Gustafson, P. (2024). The Bayes factor, HDI-ROPE, and frequentist equivalence tests can all be reverse engineered-Almost exactly-From one another: Reply to Linde et al. (2021). Psychological Methods, 29(3), 613-623. https://doi.org/10.1037/met0000507

Riesthuis, P. (2024). Simulation-Based Power Analyses for the Smallest Effect Size of Interest: A Confidence-Interval Approach for Minimum-Effect and Equivalence Testing. Advances in Methods and Practices in Psychological Science, 7(2), 25152459241240722. https://doi.org/10.1177/25152459241240722

We thank the reviewer for pointing this out. In the revised manuscript we conduct and report a thorough examination of equivalence using ROPE and Bayesian factor to support the lack of differences between regions. We did not use TOST procedures as these have low power and require huge samples be meaningful (Riesthuis, 2024). Instead we relied on Bayes factor and descriptive proportion in ROPE. Equivalence bounds and region of practical equivalence (ROPE) were defined to account for physiological variability corresponding to a small effect (Cohen’s d = 0.199, i.e. standard in equivalence testing) and axonal conduction delays between regions (i.e. ATL is further away from ‘early visual cortex’ than OCC). These analyses, now reported in the result section of the revised manuscript, along with effect sizes, confirm the hypothesis of concurrent onset latencies across VOTC.

Riesthuis P. 2024. Simulation-Based Power Analyses for the Smallest Effect Size of Interest: A Confidence-Interval Approach for Minimum-Effect and Equivalence Testing. Advances in Methods and Practices in Psychological Science 7.

Detailed methods are reported as well:

“In addition, to statistically assert whether onset latencies measured across main VOTC regions (OCC, PTL, ATL) were consistent with a concurrent (parallel) face-selective activation, we used Bayesian equivalence testing, relying on two separate metrics: (1) the percentage of differences in region of practical equivalence (ROPE), and (2) the Bayes factor using Cauchy prior. Equivalence bounds for ROPE were defined by combining two components: (1) a component of physiological variability and (2) a component reflecting expected delays in response onset between regions attributed to neural conduction delay, given the differential distances separating early visual cortex (EVC) from posterior face-selective regions (e.g. IOG) vs. anterior regions (ATL) and assuming signal mainly travels between VOTC regions through major postero-anterior axis fiber bundles of the Inferior longitudinal fasciculus (ILF) or the inferior fronto-occipital fasciculus (IFOF). Physiological variability corresponded to expected measurement noise and between-subject variability. Physiological equivalence bound was obtained by multiplying a Cohen’s d of 0.2 (conventional threshold for a negligible effect) with the (pooled) between-subjects variability in onset latency (computed for each region using a jackknife procedure and a correction factor of [N-participants – 1] to the jackknife standard deviation). For conduction delay bounds, the expected latency difference between regions under parallel activation depends on: (1) the distance between each region (considering the estimated origin of the signal is the same for all regions - EVC), and (2) neural conduction velocity. For inter-region distance we determined, for each region, the 5 and 95 percentile of the Talairach y-coordinate distribution and defined the maximal distance bounds as the distance between the y-coordinate corresponding to 5% of region 1 (e.g. OCC) to the coordinate corresponding 95% of region 2 (e.g. PTL). This resulted in the following maximum distance values: OCC-PTL: [54]mm; PTL-ATL: [54]mm; OCC-ATL: [83] mm. For conduction velocity, we used a constant value of 3.5 m/s, based on median axonal conduction velocity for cortico-cortical connections (Lemarachal et al., 2022; Van Blooijs et al., 2023), which was more conservative than using a range of values (e.g. 1.7 to 5.3 m/s based on Lemarachal et al., 2022). Maximum expected conduction delay was computed as [maximum distance / conduction speed] (e.g. for OCC to PTL: 0.054 / 3.5 = 15 ms). Resulting equivalence bounds (ROPE) were asymmetrical given than one region (e.g. OCC) is always closer to the source (EVC) than the other region (e.g. PTL) and were defined as [-1*physiologial_bound +1*physiologial_bound+max_conduction_delay]. For instance, using the z-score method to measured onset latencies, ROPE was [-12 to 27] ms for OCC to PTL, meaning that under equivalence, OCC can be activated up to 27 ms earlier than PTL (maximum conduction delay + noise), while allowing for some instances where OCC activates later (up to 12ms, due to noise only).

For each pair of region compared, we used a bootstrap procedure to (1) define the percentage of differences between regions that fall within the ROPE, (2) compute the bayes factor using a Cauchy distribution (scale = 0.5) to estimate the proportion of the prior distribution in ROPE, and the bootstrap distribution to estimate the proportion of the posterior distribution in ROPE. The bootstrap distribution was obtained using a hierarchical stratified bootstrap procedure that naturally respects the nested structure of the data, that accommodates for unequal numbers of participants, contacts, and trials across regions, as well as partially overlapping participants samples across regions.

For each bootstrap iteration, with first sample participants with replacement within each stratum (i.e. paired vs unpaired participants samples). For paired participants, sampling was performed jointly across regions to preserve the dependency structure, whereas unpaired participants were sampled independently within each region. Within each sampled participant, contacts were then resampled with replacement, and within each contact, trials were resampled with replacement. For paired participants, trial resampling was performed using identical trials across sampled contacts with a participant to preserve trial-level covariance. Resampled trials were averaged at the contact level, contact-level signals were averaged within participant, and participant-level signals were averaged to obtain a region-level response. Onset latency was then estimated from this averaged signal for each region using one of the 4 methods defined above (‘HFB response timing parameters). This procedure was repeated across 2000 bootstrap iterations to obtain a distribution of latency estimates for each region that respects the structure of data at iteration-level. Latency differences between regions were computed at each iteration, yielding a bootstrap distribution of differences which was used to compute percentage of differences in ROPE and posterior distribution for the Bayes factor.”

(4) The lack of consideration for sample sizes, the lack of pre-registration, and the lack of a method to support the null (a cornerstone of this project to demonstrate equivalence onsets between areas), suggest that the work is exploratory. This is a strength: we need rich datasets to explore, test tools and generate new hypotheses. I strongly recommend embracing the exploration philosophy, and removing all inferential statistics: instead, provide even more detailed graphical representations (include onset distributions) and share the data immediately with all the pre-processing and analysis code.

Data will be shared upon publication of the manuscript (see OSF repository in https://osf.io/2qzym). While we agree the dataset is large and could be explored in many ways, we do not consider the current study to be exploratory in nature. While our measurements could have turned out to clearly support hierarchical processing in human VOTC, our point in this manuscript is that the evidence derived from this large dataset unequivocally points instead toward concurrent activation of face-selective regions along the VOTC from IOG to antFG+ (i.e. a ~90 mm portion of cortex), with potential small variability accounted for by variability in axonal conduction velocity, signal-to-noise ratio or simple physiological variability. Other likely sources of variability such as type and density/size of fiber bundles across regions cannot easily be modeled with the current data set.

(5) Even if the work was pre-registered, it would be very difficult to calculate p-values conditional on all the uncertainty around the number of participants, the number of contacts and the number of trials, as they are random variables, and sampling distributions of key inferences should be integrated over these unknown sources of variability. The difficulty of calculating/interpreting p-values that are conditional on so many pre-processing stages and sources of uncertainty is traditionally swept under the rug, but nevertheless well documented:

Kruschke, J.K. (2013) Bayesian estimation supersedes the t test. J Exp Psychol Gen, 142, 573-603. https://pubmed.ncbi.nlm.nih.gov/22774788/

Wagenmakers, E.-J. (2007). A practical solution to the pervasive problems of p values. Psychonomic Bulletin & Review, 14(5), 779-804. https://doi.org/10.3758/BF03194105 https://link.springer.com/article/10.3758/BF03194105

All analyses and preprocessing stages are identical between regions and the number of trials is large enough not to be a constraining factor. As indicated above and below, we now report detailed equivalence testing and effect sizes and recomputed all statistics, taking into account the structure of the data as suggested by the reviewer.

(6) Currently, there is no convincing evidence in the article to clearly support the main claims.

Bootstrap confidence intervals were used to provide measures of uncertainty. However, the bootstrapping did not take the structure of the data into account, collapsing across important dependencies in that nested structure: participants > hemispheres > contacts > conditions > trials.

Ignoring data dependencies and the uncertainty from trials could lead to a distorted CI. Sampling contacts with replacement is inappropriate because it breaks the structure of the data, mixing degrees of freedom across different levels of analysis. The key rule of the bootstrap is to follow the data acquisition process, and therefore, sampling participants with replacement should come first. In a hierarchical bootstrap, the process can be repeated at nested levels, so that for each resampled participant, then contacts are resampled (if treated as a random variable), then trials/sequences are resampled, keeping paired measurements together (hemispheres, and typically contacts in a standard EEG experiment with fixed montage). The same hierarchical resampling should be applied to all measurements and inferences to capture all sources of variability. Selectivity and timing should be quantified at each contact after resampling of trials/sequences before integrating across hemispheres and participants using appropriate and justified summary measures.

The authors already recognise part of the problem, as they provide within-participant analyses. This is a very good step, inasmuch as it addresses the issue of mixing-up degrees of freedom across levels, but unfortunately these analyses are plagued with small sample sizes, making claims about the lack of differences even more problematic--classic lack of evidence == evidence of absence fallacy. In addition, there seem to be discrepancies between the mean and CI in some cases: 15 [-20, 20]; 8 [-24, 24].

In light of the reviewer’s comment, we recomputed all timing analyses using a stratified hierarchical approach to evaluate confidence intervals (using bootstrapping), statistical comparisons (using permutation tests) and equivalence testing.

This is what we wrote in the revised methods:

“The first two timing parameters of face-selective response, onset and offset latencies, were quantified per main VOTC region using a hierarchical bootstrapping approach to respect the nested structure of the data (region > participants > contacts > trials). For each bootstrap iteration and each region, we first sampled participants with replacement. Within each sampled participant, we then sampled contacts and then trials within sampled contacts, with replacement. Resampled trials, then contacts within participants, then participants within a region, were successively averaged to obtain a bootstrapped region-level response from which we derived onset latency (4 different methods) and offset latency. We obtained bootstrap distributions of onsets/offsets using 2000 bootstrap iterations per region, allowing to compute the median and 95% confidence interval for these 2 parameters.”

Then, later about permutation tests:

“Statistical significance of latency differences between main VOTC regions was assessed using a hierarchical permutation test. We use a stratification approach to partition participants into a paired set (i.e. participants that had recording contacts in the two regions compared) and unpaired set (participants with contacts in a single region). For paired participants, the region labels were randomly swapped within subject (i.e., exchanging the signals from the two regions), thereby preserving all participant-, contact-, and trial-level structure while breaking the association between region and latency estimates. For unpaired participants, participants were randomly reassigned between regions while preserving the original group sizes, to generate pseudo-groups under the null hypothesis of no regional difference. In each permutation, signals were averaged across trials, then contacts, then participants within each permuted group and latency was computed and stored from the resulting region-level signals. We performed 10000 permutations to obtain a distribution of regional differences of latencies under the null hypothesis and determine the p-value as the fraction of the null distribution larger or smaller than the observed (non-permuted) difference.”

And then about equivalence testing:

“For each pair of region compared, we used a bootstrap procedure to (1) define the percentage of differences between regions that fall within the ROPE, (2) compute the bayes factor using a Cauchy distribution (scale = 0.5) to estimate the proportion of the prior distribution in ROPE, and the bootstrap distribution to estimate the proportion of the posterior distribution in ROPE. The bootstrap distribution was obtained using a hierarchical stratified bootstrap procedure that naturally respects the nested structure of the data, that accommodates for unequal numbers of participants, contacts, and trials across regions, as well as partially overlapping participants samples across regions.

For each bootstrap iteration, with first sample participants with replacement within each stratum (i.e. paired vs unpaired participants samples). For paired participants, sampling was performed jointly across regions to preserve the dependency structure, whereas unpaired participants were sampled independently within each region. Within each sampled participant, contacts were then resampled with replacement, and within each contact, trials were resampled with replacement. For paired participants, trial resampling was performed using identical trials across sampled contacts with a participant to preserve trial-level covariance. Resampled trials were averaged at the contact level, contact-level signals were averaged within participant, and participant-level signals were averaged to obtain a region-level response. Onset latency was then estimated from this averaged signal for each region using one of the 4 methods defined above (‘HFB response timing parameters). This procedure was repeated across 2000 bootstrap iterations to obtain a distribution of latency estimates for each region that respects the structure of data at iteration-level. Latency differences between regions were computed at each iteration, yielding a bootstrap distribution of differences which was used to compute percentage of differences in ROPE and posterior distribution for the Bayes factor.”

(7) Three other issues related to onsets:

(a) FDR correction typically doesn't allow localisation claims, similarly to cluster inferences: Winkler, A. M., Taylor, P. A., Nichols, T. E., & Rorden, C. (2024). False Discovery Rate and Localizing Power (No. arXiv:2401.03554). arXiv. https://doi.org/10.48550/arXiv.2401.03554

Rousselet, G. A. (2025). Using cluster-based permutation tests to estimate MEG/EEG onsets: How bad is it? European Journal of Neuroscience, 61(1), e16618. https://doi.org/10.1111/ejn.16618

In fairness, we do not understand or share the reviewers’ concern here. Hundreds of fMRI or EEG studies use FDR or cluster tests to make inference about spatial or temporal location. We use FDR correction in one of the onset latency estimation method and only consider one-sided differences. Other methods in the revised manuscript do not use FDR correction.

(b) Percentile bootstrap confidence intervals are inaccurate when applied to means. Alternatively, use a bootstrap-t method, or use the pb in conjunction with a robust measure of central tendency, such as a trimmed mean.

Rousselet, G. A., Pernet, C. R., & Wilcox, R. R. (2021). The Percentile Bootstrap: A Primer With Step-by-Step Instructions in R. Advances in Methods and Practices in Psychological Science, 4(1), 2515245920911881.

Again, we are not sure what the reviewer’s is referring to. The confidence intervals are computed on latency estimates from bootstrapped waveforms. In the revised manuscript, these waveforms are obtained by averaging (i.e. mean) resampled trials, resampled channels, resampled participants. A trimmed mean could not be applied in this condition, except perhaps when averaging across trials. But then the trimmed mean would have to be applied separately at each time sample which would disturbed within-, or between-trial, variability.

(c) Defining onsets based on an arbitrary "at least 30 ms" rule is not recommended:

Piai, V., Dahlslätt, K., & Maris, E. (2015). Statistically comparing EEG/MEG waveforms through successive significant univariate tests: How bad can it be? Psychophysiology, 52(3), 440-443. https://doi.org/10.1111/psyp.12335

The rule of contiguous significant points is a heuristic that many researchers have used successfully to avoid spurious detection due to temporal autocorrelation. While we are aware that more sophisticated methods exist to correct for autocorrelation, such as cluster-based approaches, it is not directly usable since it requires comparing 2 conditions. The approach described in Piai et al., 2015 is interesting but incorrect as well since it relies on split-half simulations, which reduced signal-to-noise ratio, resulting in over estimated correction to be applied. In our revised manuscript, we rely on multiple methods to estimate onset latency, some of which not relying on this heuristic. Moreover, we apply plausible physiological constrains to our latency estimates, such as rejecting any onset before 40 ms after stimulus onset.

(8) Figure 5 and matching analyses: There are much better tools than correlations to estimate connectivity and directionality. See for instance:

Ince, R. A. A., Giordano, B. L., Kayser, C., Rousselet, G. A., Gross, J., & Schyns, P. G. (2017). A statistical framework for neuroimaging data analysis based on mutual information estimated via a Gaussian copula. Human Brain Mapping, 38(3), 1541-1573. https://doi.org/10.1002/hbm.23471

(9) Pearson correlation is sensitive to other features of the data than an association, and is maximally sensitive to linear associations. Interpretation is difficult without seeing matching scatterplots and getting confirmation from alternative robust methods.

We rely on Pearson correlation because this replicates the method used in Kadipasaoglu et al., 2017. It is also a widely accepted measure of (linear) relationship (in our situation we did expect linear or near linear relationships) in the literature. To address the reviewers concern, in the revised manuscript we nevertheless report, as supplementary material (Figure S10), the same functional connectivity analyses performed using the methodology and code provided in Ince et al. (2017). The results of this analyses are extremely similar to the results using Pearson’s coefficients.

**Recommendations for the authors:**

**Reviewer #1 (Recommendations for the authors):**

(1) In Figure 6, the response onset latencies are rendered in a smoothed manner on the brain surface. However, with this smoothing, variability between electrodes cannot be seen, and it would be better visualized in color, rendered in each electrode.

Latency estimates computed at individual channels are noisy, which is why we do not report individual channels latencies but rather rely on averaging signals across contiguous channels, either across whole regions (Figure 4) or across smaller volumes as in Figure 6.

(2) Onset latencies of 60 seconds seem extremely early compared to literature typically citing evoked responses with a latency of ~170ms. It would help if some additional sanity checks were shown, such as showing the latency of early visual responses. This would help with relative comparisons.

In the revised manuscript the earliest median latency is 95 ms, which is in line with previous intracranial electrophysiology literature (e.g. Jacques et al., 2016; Jacques et al., 2022 ; https://pubmed.ncbi.nlm.nih.gov/26212070/; https://pubmed.ncbi.nlm.nih.gov/36074548/). The 99% confidence intervals can result in earlier latencies both due to some participants showing early responses and noise in latency estimates. Also please keep in mind that latency estimates are usually earlier when combining data across channels/participants compared to individual channels simply due to differences in SNR or across participants (see e.g. Kadipasaoglou et al., 2017).

The reviewer indicates “…to literature typically citing evoked responses with a latency of ~170ms.”. We are assuming that they refer to the face-selective N170 ERP component measured on the scalp in EEG. Even with this ERP component, the face-selective response usually starts around 120-130 ms after stimulus onset (e.g. Rousselet et al., 2008; Jacques, Retter and Rossion, 2016; https://pubmed.ncbi.nlm.nih.gov/18831616/; https://pubmed.ncbi.nlm.nih.gov/27138205/) at scalp level. With the same highly sensitive paradigm as used here in EEG, we have systematically shown latency onsets of face-selective activity shortly after 100 ms (e.g., Retter et al., 2020; also Quek & Rossion, 2017) not accountable for by low-level visual cues (i.e., not present for phase-scrambled stimuli; Rossion et al., 2015; Or et al., 2019). Our latency onsets are also in line with spiking activity recorded with the same approach in the LatFG (Laurent et al., 2026) https://papers.ssrn.com/sol3/papers.cfm?abstract_id=5955677

(3) Figure 6B shows that the variability of latencies in the ATL is larger than the variability of latencies in the PTL. It would be helpful to evaluate whether, rather than in mean onset latencies, there is a change in variability in onset latency along the VOTC.

This is an interesting point. However, it is difficult to evaluate since SNR is reduced in the ATL compared to OCC or PTL (Jacques et al., 2022). As a result, any measured modulations in the variability of onset latencies along VOTC may simply reflect changes in the precision of latency estimation driven by SNR variability.

(4) Line 415 typo: 'there appears to be no delay' instead of 'there appear to be no delay'.

We thank the reviewer for their careful reading of our manuscript. This has been corrected.

(5) The discussion states in lines 455-457 that "a large proportion of neuronal populations in anterior VOTC regions exhibiting similar activity to different face images independently of the context in which they appear". However, this claim about similar activity to different faces should be evaluated and tested at the single-trial level. In addition, it is not clear how context was varied in the experimental design.

Context is variable because each face image appears directly after a different object image (or object images) in the sequence. We have shown also in previous studies with this paradigm in EEG that the time-course of face-selective responses is similar across base frequencies (3-15 Hz) unless the rate is too fast, and whether an orthogonal or explicit face categorization task is used (Retter et al., 2020; https://pubmed.ncbi.nlm.nih.gov/32119982/). Note that we do not claim that activity is identical across images but similar – if it was not (largely) similar, the averaged response would be jittered and low.

(6) Line 516-517: DTI does not provide evidence for whether connectivity is direct or not, and what the directionality of connectivity is between two areas. This sentence should therefore state "..., suggest independent connections between early visual cortex and face-selective regions...".

This has been rephrased.

(7) Line 555 in the discussion, the definition of low-level visual should be expanded to include other early visual areas that have been demonstrated to respond earlier than VOTC (e.g. Martin et al., 2019, JNeurosci https://doi.org/10.1523/JNEUROSCI.1889-18.2018), to avoid the suggestion that V1 directly projects synaptically to all of VOTC (e.g. Markov et al., 2014, Cerebral Cortex, https://doi.org/10.1093/cercor/bhs270).

We are not proposing that V1 directly projects directly/synaptically to all of VOTC, i.e., without other low-level retinotoptic areas involved; only that face-selectivity in the association cortex is not organized hierarchically. We have revised this sentence.

(8) Line 585, for the sentence: "with temporal synchrony strengthening their connections", evidence or citations should be provided.

Citations have been provided.

(9) It is not clear what is meant in the paragraph starting in line 571: do the authors suggest that top-down signals are not necessary for fast recognition of clear views of faces, or additionally argue that these top-down signals are not necessary for detecting ambiguous or degraded inputs as faces?

Exactly: That top-down (i.e., descending) signals may contribute but would not be necessary for fast recognition of clear views of faces AND for detecting ambiguous or degraded inputs as faces.

(10) No statement was provided on data or code availability.

Data will be made available on a repository upon publication (see https://osf.io/2qzym).

**Reviewer #2 (Recommendations for the authors):**

(1) FDR correction: which one? Please provide a reference.

We now provide a reference, both in the results and methods: Benjamini and Hochberg, 1995.

(2) In the introduction, this statement is too strong: "arguably the most familiar and ecologically valid stimulus". It is unclear how static 2D representations of faces are the most familiar and valid stimuli. Could you rephrase this? What about other very familiar stimuli like letters, words and biological motion?

This statement is not about static 2D images of faces, but faces in general (in their natural environment). We do consider human faces (in general, not restricted to laboratory context) to be indeed the most familiar and ecologically important stimulus, both from an ontogenetic and phylogenetic perspective, unlike written material.

(3) About the questioning of a strict temporal hierarchy, this EEG reference comes to mind: Foxe, J. J., & Simpson, G. V. (2002). Flow of activation from V1 to the frontal cortex in humans. Experimental Brain Research, 142(1), 139-150. https://doi.org/10.1007/s00221-001-0906-7

As confirmed by Foxe et al. ’s (2002) paper to which the reviewer is referring to, there is indeed ample evidence that areas in the dorsal stream or frontal cortex (e.g. FEF) are activated very soon after V1 and before many ventral stream regions (e.g. Lamme and Roelfsema, 2000; https://pubmed.ncbi.nlm.nih.gov/11074267/). While Foxe et al.’s 2002 is highly valuable, it can hardly be compared with our current study which looks specifically into ventral stream areas which are largely indistinguishable using scalp EEG as in Foxe et al. ’s paper.

(4) Regarding statistical significance, there is no such thing as a "trend". The threshold for a trend should have been pre-registered and applied to both sides of the magical boundary, for instance, with matching conclusions for a "trend toward non-significance (p=0.04)". P values near 0.05 provide weak support against the null. I would suggest leaving it at that. Nothing special happens at 0.05.

This no longer appears in the revised manuscript.
